# Predictors of Diarrhea after Hepatectomy and Its Impact on Gastrointestinal Quality of Life in Living Donors

**DOI:** 10.1371/journal.pone.0166576

**Published:** 2016-11-18

**Authors:** Szu-Han Wang, Ying-Zi Ming, Ping-Yi Lin, Jiun-Yi Wang, Hui-Chuan Lin, Chia-En Hsieh, Ya-Lan Hsu, Yao-Li Chen

**Affiliations:** 1 Organ Transplant Center, Changhua Christian Hospital, Changhua, Taiwan; 2 Transplantation Center, Third Xiangya Hospital of Central South University, Changsha, China; 3 Transplant Medicine & Surgery Research Centre, Changhua Christian Hospital, Changhua, Taiwan; 4 Department of Health Care Administration, Asia University, Taichung, Taiwan; 5 Department of Senior Citizen Welfare and Business, Hung Kuang University, Taichung, Taiwan; 6 Department of General Surgery, Changhua Christian Hospital, Changhua, Taiwan, and School of Medicine, Kaohsiung Medical University, Kaohsiung, Taiwan; Kaohsiung Medical University Chung Ho Memorial Hospital, TAIWAN

## Abstract

**Background:**

Donor safety and preservation of donor health after living liver donation are of paramount importance. Diarrhea has a significant influence on gastrointestinal quality of life among donors who have undergone living donor hepatectomy. Thus, we aimed to investigate predictors of diarrhea after hepatectomy and its impact on gastrointestinal quality of life in living donors.

**Methods:**

We retrospectively examined the medical records of 204 living liver donors who underwent hepatectomy during the period January 2010 to June 2013 at a single medical center. Diarrhea was defined as the passing of three or more liquid stools per day. The Chinese version of the Gastrointestinal Quality of Life Index (GIQLI) was used to assess the influence of diarrhea on quality of life in donors.

**Results:**

During the study period, diarrhea was diagnosed in 62 (30.3%) of the 204 donors and the duration of diarrhea in the majority of them (n = 46, 74%) was <12 months. Risk factors associated with diarrhea included age [risk ratio (RR) = 0.84, 95% confidence interval (CI): 0.79–0.89, risk difference = 16%], and chronic cholecystitis (RR = 0.48, 95% CI: 0.24–0.99, risk difference = 52%). Compared to donors without diarrhea, donors with diarrhea had lower GIQLI scores in the following GIQLI domains: GI symptoms (1.8 vs. 3.6), physical function (2.1 vs. 3.5), emotional function (3.0 vs. 3.6), social function (3.3 vs. 3.7), and treatment reaction (2.6 vs. 3.7).

**Conclusions:**

Our findings show that younger donors and those without chronic cholecystitis are at increased risk for diarrhea after living donor hepatectomy and that diarrhea is associated with lower GIQLI scores after hepatectomy.

## Introduction

The frequency with which living donor liver transplantation (LDLT) is performed has increased markedly in the last few decades because of the severe shortage of cadaveric donor organs. Adult-adult right hepatic lobe LDLT is one of the most effective means of allocating a liver graft to a recipient with end-stage liver disease. However, the procedure exposes the living donor to the risk of a number of complications including biliary complications, abdominal discomfort and infection. In order to minimize the risk, all aspects of donor outcomes must be measured to determine the impact of donation. [1–3]

Although the mortality rate among donors is relatively low (0.2%), the donor morbidity rate is as high as 59%.[2,4–6] The incidence of gastrointestinal complications is also high (53%) after living donor hepatectomy (including cholecystectomy), with diarrhea being the most common symptom (31%).[7] Cholecystectomy is routinely performed at most transplant center during living donor hepatectomy.[8] The incidence of diarrhea after laparoscopic cholecystectomy incidence is around 25%, and younger age was has been shown to be independently associated with the development of post cholecystectomy diarrhea. [9]

Although many studies on life quality in living donors after hepatectomy have been conducted, risk factors associated with postoperative diarrhea as well as gastrointestinal quality of life after liver donation have not been investigated. In this retrospective study, we tried to determine the factors that are predictive of postoperative diarrhea as well as its impact on gastrointestinal quality of life in living liver donors after hepatectomy.

## Materials and Methods

### Donors

**Fig 1:**
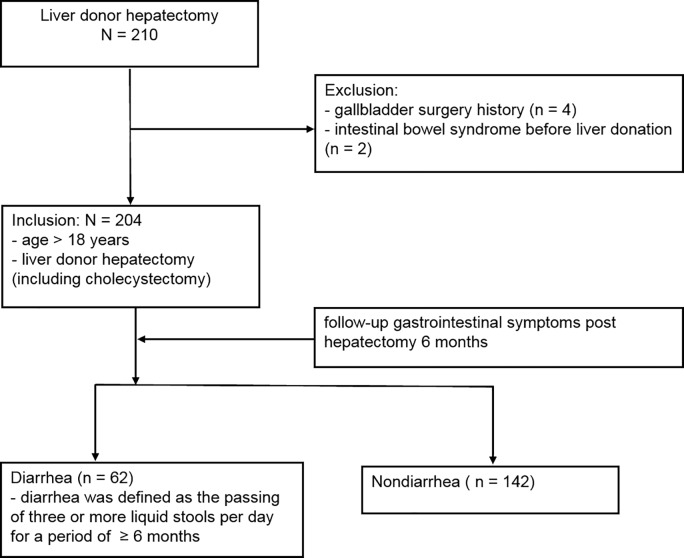
Screening and enrollment.

We retrospectively enrolled living liver donors who underwent hepatectomy during the period January 2010 to June 2013 at the Changhua Christian Hospital. The inclusion criteria included donors aged > 18 years who underwent liver donor hepatectomy (including cholecystectomy). Donors with a history of gallbladder surgery (N = 4) and those with intestinal bowel syndrome before liver donation (N = 2) were excluded. Therefore, a total of 204 donors fulfilled the inclusion criteria and were included in the analysis (Fig 1). The protocol was approved by the institutional review board of the Changhua Christian Hospital and the study was conducted in accordance with the Declaration of Helsinki. (Document no. 140708). Written informed consent was obtained from all donors.

### Study Process

In general, donors would routinely return to the clinic after hepatectomy and were inquired about their diarrhea status in each return. They were also invited to fill up the Chinese version of the Gastrointestinal Quality of Life Index (GIQLI) questionnaire at the time point of 6 months after hepatectomy.

Diarrhea was defined as the passing of three or more liquid stools per day for a period of at least 6 months.[10,11] In this study, we stratified donors into a diarrhea group or a non- diarrhea group. The time of diarrhea group from liver donation to administration of the gastrointestinal symptoms was 24 months (examined medical records during 6–24 month).

### Clinical data

Donor characteristics included gender, age, body mass index (BMI, Kg/m^2^), donor-recipient relationship t, remnant liver volume (RLV, %), hepatic steatosis (as determined on abdominal computed tomographic image) and presence of chronic cholecystitis. Chronic cholecystitis, which was established based on radiologic evidence of a thickened gallbladder wall and histologic evidence of chronic inflammation and fibrosis [12], duration of surgery (min), the need for second hospitalization (for morbidity), ability to return to pre-donation job and duration of diarrhea (months).

The GIQLI is a 36-item questionnaire designed to assess the impact of gastrointestinal (GI) symptoms and disease on daily life. The questionnaire measures the following five domains: GI symptoms (19 items), physical function (7 items), emotional function (5 items), social function (4 items), and treatment reaction (1 item). Each item is scored from 0 to 4 (0 being the worst and 4 the best condition), the range of the total score was 0–144.[13] To cope with Taiwan population, the Chinese (Taiwan) version of the GIQLI was used for this study. This version of GIQLI has been shown to have good test-retest reliability (r = 0.92, p = 0.001) and internal consistency (Cronbach's alpha = 0.92) and has been found to significantly correlate with the Chinese (Taiwan) version of the generic 36-item Short-Form Health Survey (SF-36) which is commonly used in assessing generic quality-of-life.[14]

### Statistical Analysis

Continuous variables are presented as mean ± standard deviation (SD) and categorical variables are presented as percentages. The Mann-Whitney U-test was used to compare continuous variables between donors with diarrhea and those without the symptom. The chi-square test was used to compare differences in categorical variables between the two groups. Significant variables in the univariate analyses were then included in a multiple logistic-regression model to identify the most important risk factors for diarrhea after LDLT. A P-value <0.05 was considered to represent statistical significance. All statistical analyses were performed on a personal computer with the statistical package SPSS for Windows (Version 18, SPSS, Chicago, Il, USA).

## Results

### Donor Characteristics

A total of 204 donors (mean age, 29.8 ± 8.1 years) met the inclusion criteria and 51.1% of them were men. The mean BMI was 22.9 ± 3.4 Kg/m^2^ and 87.3% of the donors were immediate family members of the recipient (Table 1).

**Table 1:** Characteristics of living liver donor.

Characteristics ^a^	Diarrhea (n = 62)	Nondiarrhea (n = 142)	Total (N = 204)	p-value
Age	24.3 ± 4.4	32.1 ± 8.2	29.8 ± 8.1	0.001
Male ^b^	33 (53.2)	72 (50.7)	105 (51.1)	0.763
BMI	22.4 ± 3.6	23.2 ± 3.4	22.9 ± 3.4	0.138
**Relationship with recipient** ^b^				
Spouse	3 (4.8)	16 (11.3)	19 (9.3)	0.204
Immediate family	58 (93.6)	120 (84.5)	178 (87.3)	
Other	1 (1.6)	6 (4.2)	7 (3.4)	
RLV (%)	39.4 ± 5.2	38.3 ± 5.0	38.6 ± 5.1	0.146
Hepatic steatosis ^b^	9 (14.5)	45 (31.7)	54 (26.5)	0.010
Chronic cholecystitis ^b^	19 (30.6)	81 (57.0)	100 (49.0)	0.001
Duration of surgery (min)	253 ± 45	248± 61	249 ± 56	0.512
Second hospitalization ^b^	6 (9.7)	8 (5.6)	14 (6.9)	0.367
Returned to pre-donation job ^b^				
<3(month)	27 (43.5)	88 (62.0)	115 (56.4)	0.021
3–6	34 (54.8)	53 (37.9)	87 (43.0)	
>6	1 (1.7)	1 (0.01)	2 (1.0)	
Duration of diarrhea				
≤12(month)	46 (74.0)			
>12–24	12 (19.5)			
>24	4 (6.5)			
**GIQLIS**				
Gastrointestinal symptoms	1.8 ± 0.4	3.6 ± 0.5	3.0 ± 0.9	<0.001
Physical function	2.1 ± 0.4	3.5 ± 0.6	3.1 ± 0.8	<0.001
Emotional function	3.0 ± 0.5	3.6 ± 0.4	3.5± 0.6	<0.001
Social function	3.3 ± 0.4	3.7 ± 0.4	3.6 ±0.5	<0.001
Treatment reaction	2.6 ± 0.5	3.7 ± 0.4	3.4± 0.7	<0.001

^a^ Data are shown as mean ± standard deviation and compared using the Mann-Whitney test.

^b^ Data are shown as n (%) and compared using the chi-square test.

BMI = body mass index, RLV = remnant liver volume, GIQLIS = Gastrointestinal Quality of Life Index score

Of the 204 donors, diarrhea was diagnosed in 62 (30.3%) after liver donation. Donors with diarrhea were significantly younger (24.3 vs. 32.1 years) (p = 0.001), had a lower rate of hepatic steatosis (14.5% vs. 31.7%) (p = 0.010) and had a lower rate of chronic cholecystitis (30.6% vs. 57.0%) (p = 0.001) than donors without diarrhea. In the majority (74%) of donors with diarrhea, the duration was ≤12 months. Approximately 50% of donors who developed diarrhea returned to their pre-donation job within 3–6 months whereas 62% of donors without diarrhea returned to work in less than 3 months after liver donation (p < 0.05). (Table 1)

### Gastrointestinal Quality of Life

Compared to donors without diarrhea, donors with diarrhea had lower GIQLI scores in the following GIQLI domains (p < 0.001): GI symptoms (1.8 vs. 3.6), physical function (2.1 vs. 3.5), emotional function (3.0 vs. 3.6), social function (3.3 vs. 3.7), and treatment reaction (2.6 vs. 3.7) (Table 1).

### Predictors of diarrhea after living donor hepatectomy

Univariate analysis revealed that age [risk ratio (RR) = 0.83, 95% confidence interval (CI): 0.78–0.88], hepatic steatosis (RR = 0.36, 95% CI: 0.16–0.80), and chronic cholecystitis (RR = 0.33, 95% CI: 0.17–0.62) were factors associated with diarrhea after living donor hepatectomy. Multivariate analysis of those factors revealed that age (RR = 0.84, 95% confidence interval (CI): 0.79–0.89, risk difference = 16%) and chronic cholecystitis (RR = 0.48, 95% CI: 0.24–0.99, risk difference = 52%) (Table 2) were risk factors for the development of postoperative diarrhea.

**Table 2:** Associated factors of diarrhea after living liver donor hepatectomy in living liver transplant donor.

	Univariate analysis				Multivariate analysis			
Factor	risk ratio	95% CI	risk difference (%)	p	risk ratio	95% CI	risk difference (%)	p
Age	0.83	0.78–0.88	17	<0.001	0.84	0.79–0.90	16	<0.001
Hepatic steatosis	0.36	0.16–0.80	64	0.013	0.74	0.29–1.86	26	0.525
Chroniccholecystitis	0.33	0.17–0.62	67	0.001	0.48	0.24–0.99	52	0.048

## Discussion

Living donor liver transplantation is a relatively safe procedure with a low mortality rate; however, donors are at risk of developing a number of post-procedural complications including biliary complications, infections and gastrointestinal complications, of which diarrhea is one of the most common, occurring in approximately 30% of patients with GI complications.[7,15] Studies have shown that chronic diarrhea not only results in a decline in but also markedly reduces the donor’s quality of life.[7,15] In our study, 30.3% (62/204) of donors developed new-onset diarrhea after hepatectomy, which is in agreement with previous findings in living liver donors.[8] Removal of a normal gallbladder can lead to several functional disorders with potential injury to the donors.[8] After cholecystectomy, the bile is released continuously into the intestinal tract, and such dyspeptic symptoms as abdominal distention and diarrhea are observed after intake of high-fat diets, because of no or insufficient bile in the intestinal tract.[8] Cholecystectomy shortens the gut transit time by accelerating passage through the colon and diarrhea develop early and persist for at least 4 years after cholecystectomy.[16] All donors in this study received nutritional consultations by a dietitian before surgery. Dong et al reported that diarrhea occurring 1 week after cholecystectomy correlates with the consumption of a low-fat diet (β = -0.177, p = 0.000) and a preoperative diarrhea scale (β = 0.311, p = 0.031). [9] However, the authors of that study did not specify the duration for which donors should follow a low-fat diet after surgery. Therefore, all donors in our study were advised to follow a low-fat diet for 3 months to reduce the possibility of diarrhea after living donor hepatectomy.

In this study, the incidence of post-hepatectomy diarrhea in living liver donors was 30.3% and diarrhea resolved in most donors within 24 months. We found that younger age and lack of chronic cholecystitis were independently associated with the development of postoperative diarrhea. Our findings are similar to those reported in studies on postoperative outcomes in patients who underwent laparoscopic cholecystectomy (LC), namely that young patients with a high body mass index were at risk of developing chronic diarrhea after surgery.[9] Fisher et al. reported that young age (<50 years) alone or in combination with overweight (≥25 Kg/m^2^) or obesity (≥30 Kg/m^2^) with associated with 4.2- to 11.1- fold increase in the odds of developing post LC diarrhea.[17] In this study, 69.7% (142/204) of donors did not develop diarrhea after liver donation. Of them, 31.7% had hepatic steatosis and 53.5% had chronic cholecystitis. All living donors have been evaluated to ensure they have met criteria for liver donation and without any significant medical problems before have living liver hepatectomy. Although previous studies found higher BMI and hepatic steatosis may contribute to the development of postoperative diarrhea [17], we found that neither of those variables were predictive of diarrhea after donor hepatectomy. These inconsistent findings are most likely due to the limited sample size and selection criteria. For example, the mean BMI in the two groups of donors was within the normal range (22.4 v.s. 23.2 Kg/m2), indicating that few if any of the donors were overweight or obese. In addition, we did not consider the severity of steatosis when selecting donors for inclusion in the study. This may cause inconsistent findings.

Sotiropoulos et al. found that donors with chronic cholecystitis before liver donation who developed persistent diarrhea after cholecystectomy were better able to tolerate foods with a high fat content, resulting in fewer diarrhea-related complications.[2] Moreover, studies have shown that there is a positive correlation between hepatic steatosis and chronic cholecystitis.[18,19] Predictors of diarrhea after living donor hepatectomy (including cholecystectomy) has not been previously reported, even in instances of liver trauma or oncologic resection where removal of a “normal” gallbladder occurred for technical reasons.

Most of the donors in this study returned to work within 6 months after surgery. Among donors with diarrhea, 54.8% returned to their pre-donation job within 3–6 months after surgery and 62% of those without diarrhea returned to work less than three months after surgery. Trotter et al. reported that donor feel poor perceived physical function (abdominal discomfort, diarrhea, loss of appetite,nausea, trouble concentrating, poor appetite, weakness, difficulty sleeping) after hepatectomy, when donor physical function recovery, they returned to pre-donation job mean time about 2.4–6 month.[20]

Wanjura et al. reported that the most common gastrointestinal symptoms after cholecystectomy were diarrhea, bowel urgency and flatus, and that GIQLI scores in those patients were markedly lower than those in the general population.[21] In this study, we found that donors who developed postoperative diarrhea had lower GIQLI scores and lower scores in the physical function, emotional function, social function, and treatment reaction domains than donors who did not develop diarrhea, which might explain why donors with postoperative diarrhea returned to work later than donors who did not develop diarrhea after donor hepatectomy.

The present study has some limitations. Firstly, diarrhea status of a patient was self-reported. Self-reported data were generally reliable, however biases due to incorrect recall or unwilling to reveal personal information are unavoidable. Secondly, the data were collected from a single medical center in Mid-Taiwan, and this may somewhat limit the applicability of the study results. Larger scale studies are required to further verify the findings of the present study.

## Conclusions

The incidence of post-hepatectomy diarrhea in living donors was 30.3% in this study. We found that younger age and lack of chronic cholecystitis were independently predictive of diarrhea after living donor hepatectomy. Diarrhea in most donors resolved spontaneously within 24 months. Diarrhea was associated with lower GIQLI scores after donor hepatectomy, indicating that gastrointestinal quality in living donors should be monitored over the long-term after living donor liver transplantation.
